# Influence of Asphalt Emulsion Inclusion on Fly Ash/Hydrated Lime Alkali-Activated Material

**DOI:** 10.3390/ma14227017

**Published:** 2021-11-19

**Authors:** Thanon Bualuang, Peerapong Jitsangiam, Teewara Suwan, Ubolluk Rattanasak, Weerachart Tangchirapat, Suriyah Thongmunee

**Affiliations:** 1 Graduate Program in Civil Engineering, Department of Civil Engineering, Faculty of Engineering, Chiang Mai University, Huay Kaew Road, Muang, Chiang Mai 50200, Thailand; thanon_bualuang@cmu.ac.th; 2 Center of Excellence in Natural Disaster Management, Department of Civil Engineering, Faculty of Engineering, Chiang Mai University, Huai Kaew Road, Mueang, Chiang Mai 50200, Thailand; teewara.s@cmu.ac.th (T.S.); suriyah@eng.cmu.ac.th (S.T.); 3 Department of Chemistry, Faculty of Science, Burapha University, Chonburi 20131, Thailand; ubolluk@go.buu.ac.th; 4 Construction Innovations and Future Infrastructure Research Center (CIFIR), Department of Civil Engineering, Faculty of Engineering, King Mongkut’s University of Technology Thonburi, Bangkok 10140, Thailand; weerachart.tan@kmutt.ac.th

**Keywords:** asphalt emulsion, pozzolanic material, alkali-activated binder, cementless materials, fly ash, hydrated lime

## Abstract

Supplementary cementitious materials have been widely used to reduce the greenhouse gas emissions caused by ordinary Portland cement (OPC), including in the construction of road bases. In addition, the use of OPC in road base stabilization is inefficient due to its moisture sensitivity and lack of flexibility. Therefore, this study investigates the effect of hybrid alkali-activated materials (H-AAM) on flexibility and water prevention when used as binders while proposing a new and sustainable material. A cationic asphalt emulsion (CAE) was applied to increase this cementless material’s resistance to moisture damage and flexibility. The physical properties and structural formation of this H-AAM, consisting of fly ash, hydrated lime, and sodium hydroxide, were examined. The results revealed that the addition of CAE decreased the material’s mechanical strength due to its hindrance of pozzolanic reactions and alkali activations. This study revealed decreases in the cementitious product’s peak in the x-ray diffraction analysis (XRD) tests and the number of tetrahedrons detected in the Fourier transform infrared spectroscopy analysis (FTIR) tests. The scanning electron microscope (SEM) images showed some signs of asphalt films surrounding hybrid alkali-activated particles and even some unreacted FA particles, indicating incomplete chemical reactions in the study material’s matrix. However, the H-AAM was still able to meet the minimum road base strength requirement of 1.72 MPa. Furthermore, the toughness and flexibility of the H-AAM were enhanced by CAE. Notably, adding 10% and 20% CAE by weight to the hybrid alkali-activated binder produced a significant advantage in terms of water absorption, which can be explained by its influence on the material’s consolidation of its matrices, resulting in significant void reductions. Hence, the outcomes of this study might reveal an opportunity for developing a new stabilizing agent for road bases with water-prevention properties and flexibility that remains faithful to the green construction material concept.

## 1. Introduction

At present, ordinary Portland cement (OPC) replacements are being developed to reduce greenhouse gas emissions in the construction industry. Many studies have revealed the benefits of supplementary cementitious materials (SCMs), such as industrial fly ash and hydrated lime, when replacing some OPC in concrete, including cost reductions and fewer greenhouse gas emissions [1,2,3,4,5]. Many SCMs incorporating green materials, i.e., geopolymers and alkali-activated materials (AAMs), have been introduced. These silica- and alumina-rich materials are activated using alkali solutions, such as sodium hydroxide (NaOH) and sodium silicate (Na_2_SiO_3_), resulting in new and green construction materials with mechanical properties equal to the general concrete for construction [6,7,8,9]. Theoretically, pozzolanic materials with appropriate alkali activators can form the cementitious products calcium aluminosilicate hydrate gel (C-A-S-H), calcium silicate hydrate gel (C-S-H), and sodium aluminosilicate hydrated product (N-A-S-H) [10,11,12,13,14]. These structural formations are similar to the hydration product resulting from mixing OPC and water [15]. Some formations with lower mechanical strengths than cement-based materials have been used in road bases and subbase structural layers. Consequently, the AAMs and pozzolanic materials have been used as replacements for OPC in this specific work. However, it is still necessary to increase their long-term durability for actual road conditions.

AAMs and pozzolanic materials are suitable under compressive loading conditions. However, they are probably prone to more brittleness and less waterproofness than necessary for roadway construction materials. During the rainy season, the relatively high water can establish premature cracks in road pavement structures, allowing liquids to access and penetrate capillary and air voids, decreasing the durability of cement-based materials and AAMs [16,17,18]. Stabilizing materials with high moisture sensitivity decrease stiffness caused by excess moisture from an external environment [19,20], negatively impacting crucial pavement properties [21,22]. This problem results in redundant maintenance costs and cannot be resolved in many countries. Hardiness, flexibility, and waterproofness are necessary for long-term service. For this reason, a comprehensive solution for the brittleness and lack of waterproofness in cement and SCMs is necessary for attaining long-term durability.

Asphalt emulsion (AE), a stabilized asphalt by a chemical emulsifier, is an exciting additive due to the efficient elasticity and waterproofness it brings to cement-based materials and AAMs [23,24,25,26]. Researchers have investigated and applied AE as an additive in concrete or mortar and correlated its addition to dynamic track slabs and ballasts, among other structures [27,28]. Some studies have found that it can improve pavement engineering properties in asphalt-treated base materials by increasing flexibility, resilience, and durability [29,30,31,32]. Moreover, the workability of AE in cement-based material influences the setting process [33,34], which may be appropriate for pavement constructions requiring a reasonable period of field-mixing preparation. Due to its inherent good flexibility and moisture resistance, AE could be used to enhance the flexibility and waterproofness of chemically stabilized road pavement materials.

The interaction mechanism of a cement-AE-based material causes asphalt droplets with electrostatic charges to form an emulsifier that can react with the hydration product, such as Portlandite and C-A-S-H gel, by exchanging the polar from these different chemical components [35]. The asphalt molecules converge around the cement paste matrices and form a stiff film. The cement particles absorb the water in AE, leading to the reduced spaces in the pastes’ matrices and reduced voids [26,36,37]. However, the hydration of cement-based materials is delayed due to the asphalt droplets covering the cement particles, resulting in incomplete primary hydration with water. This retarding effect can increase the initial and the final setting times, allowing AE to significantly retard cement hydration at a high AE to cement ratio [38]. In addition, hydration heat is blocked by thin films of AE on the cement particles, resulting in decreases in mechanical strength and stiffness [39,40,41]. No new crystalline substances or chemical functional groups have been found during the interaction process between cement and the AE [42]. However, based on the previous studies, there was almost no study of a hybrid alkali-activated material (H-AAM) prepared with fly ash (FA), hydrated lime (HL), and solid sodium hydroxide (NaOH); and the effects of adding AE into such material is still questionable. Even FA itself has toxicity manners from leaching [43,44]; the FA used in this study is from the common source of FA used in the concrete industry in Thailand. Therefore, it is categorized as a standard constituent of the cementitious mixing material.

Therefore, this study aimed to investigate the influence of AE on the mechanical properties and structural formations of such the H-AAM. The AE was added to the mixture in varying amounts. The mechanical characteristics of workability, compressive and flexural strength, water absorption, and shrinkage were examined. Meanwhile, the resulting mixtures’ morphologies and microstructures were analyzed via x-ray diffraction (XRD), Fourier transform infrared spectroscopy (FTIR), scanning electron microscope (SEM), and pore size distribution tests.

## 2. Preliminary Study of the H-AAM for Road Bases

**Figure 1:**
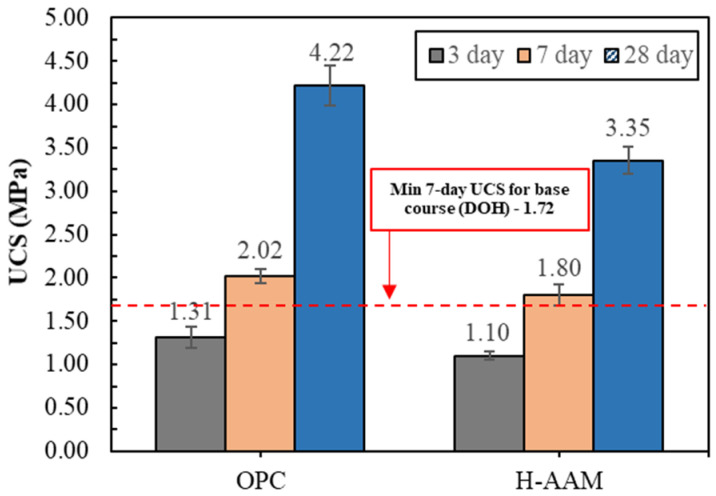
UCS results of road base materials stabilized by Portland cement and the H-AAM obtained during the preliminary study.

Preliminary research conducted at Chiang Mai University, Thailand, established that the H-AAM could be used as a stabilizing agent in road bases. The H-AAM and Portland cement (as a benchmark) were utilized as stabilizing agents, while the crushed rock was used as the parent material [45]. From Figure 1, it can be seen that the H-AAM achieved the target unconfined compressive strength (UCS) required by the Department of Highways, Thailand, on day 7 [46,47]. These UCS values showed that the H-AAM could be used to achieve the minimum strength required, even though its strength was less than that of the OPC-based material. Therefore, this H-AAM could be utilized as an OPC replacement in road stabilization. This study also explored the influence of AE on the flexibility and waterproofness of this material.

## 3. Materials and Methods

### 3.1. Materials and the Mix Design

**Figure 2:**
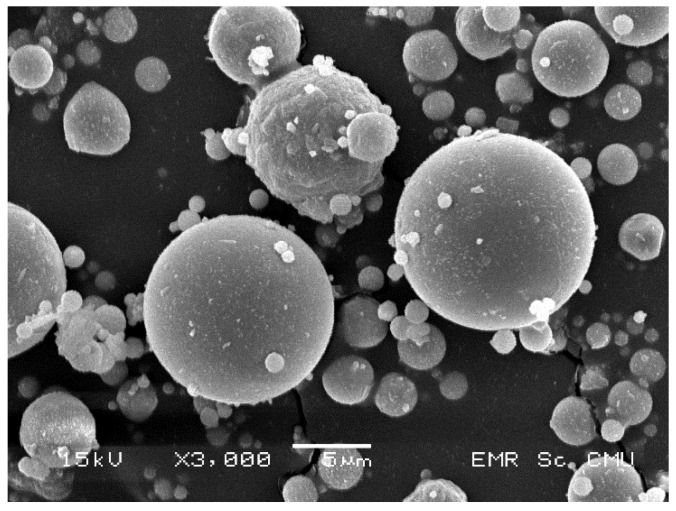
The SEM images of the FA in this study.

FA and HL were used as raw materials in the production of the alkali-activated material. The FA was obtained from the Mae Moh power plant, Lampang Province, Thailand. Meanwhile, the HL came from the limestone quarry in Saraburi Province, Thailand. Figure 2 illustrates the morphology of the FA observed by using SEM (JEOL JSM-5910LV, gold coated, WD: 11 mm, EHT: 15 kV, JEOL Inc., Pleasanton, CA, USA). Table 1 shows the oxide compositions of the raw materials obtained with X-ray fluorescence (XRF, JEOL JSX3400R, JEOL Inc., Pleasanton, CA, USA). The FA was classified as a high-calcium or class C fly ash with significant CaO content (24.83%). Apart from the CaO, the FA had high contents of SiO_2_ (32.47%), Al_2_O_3_ (15.84%), and Fe_2_O_3_ (14.64%). The specific gravity and Blaine fineness of the FA were 2.11 and 2400 cm^2^/g, respectively.

Meanwhile, the HL contained CaO compounds with high loss on ignition (LOI). High LOI can imply the evaporation of water and decomposition of Ca(OH)_2_ at 512 °C. The specific gravity and blain fineness of HL were 2.16 and 1600 cm^2^/g, respectively. Commercial-grade NaOH at 98% purity was used as an alkali activator. The NaOH was ground finely (approximately 2.00 mm particles) before being added to the mixtures.

In this study, the slow-setting CAE (CSS-1h) used as an additive was provided by the Asian Asphalt Co. of Thailand. The CAE with a positive electrostatic charge was suspended in hydrochloric acid solution, containing 67.3% solid residue asphalt by total modified asphalt weight. The properties of the CAE are shown in Table 2.

**Figure 3:**
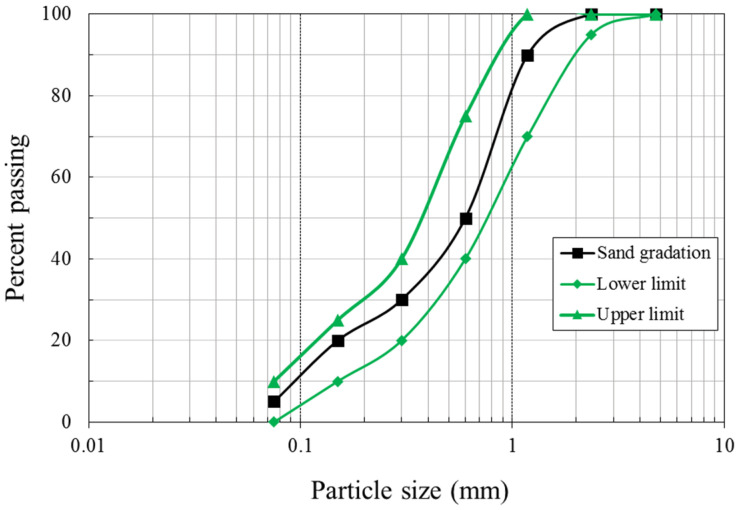
Sand gradation used for mortar samples.

Based on Table 3, the hybrid alkali-activated mixtures with different CAE contents were prepared using the binder-sand ratio (b/s) of 1.00:2.75, in which the sand with specific gravity was 2.65 and the gradation shown in Figure 3. The control mixture as the H-AAM sample consisted of FA, HL, and NaOH, following Table 3. According to the previous study, the constituents of the H-AAM binder was validated the potential on cement replacement in road base construction [45]. Proportions of CAE to the hybrid binder of 0, 0.05, 0.10, and 0.20 were used in conjunction with the added water was water-to-binder ratio (w/b) of 0.45:1.00. For the microstructure and structural formation analyses, hybrid alkali-activated pastes with different CAE contents were prepared.

During the mixing procedure, the dry binders were added to the saturated sand and mixed homogeneously. Subsequently, the water was added, followed by the CAE. The resulting materials were then mixed for 180 s with a Hobart mixer.

### 3.2. Methodology and Experiments

**Figure 4:**
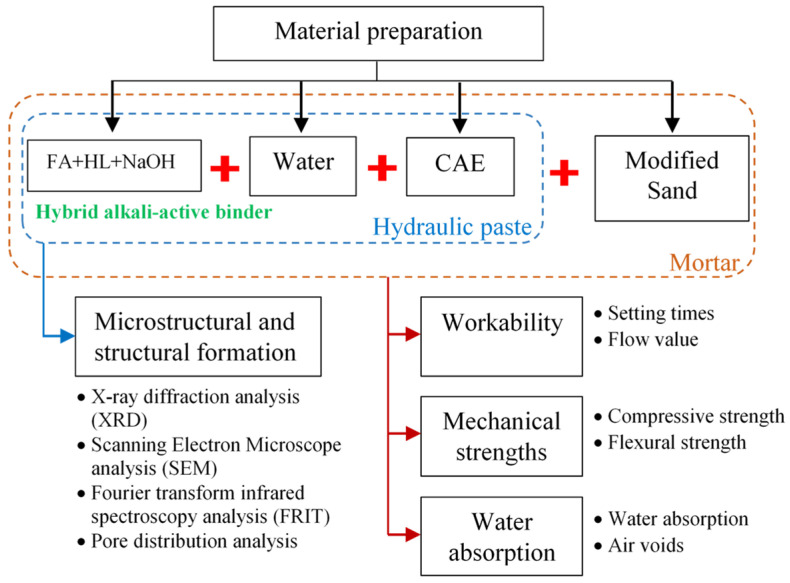
Perspective diagram of the research.

Figure 4 presents the perspective diagram for the research to clarify the preparation of the materials and the experiments. Mortar samples were examined to obtain the physical properties of the target specimens. Meanwhile, microstructural and structural formation analyses were performed on the hydraulic pastes.

In this study, the binder combined with varying CAE contents was examined. The strength, water absorption, elasticity, and toughness of the resulting materials were determined. All test specimens were prepared in fresh mortar form. Furthermore, microstructural analyses were conducted to support all experimental test results.

#### 3.2.1. Flow and Setting Time Tests

Mortar samples were investigated for their workability within 90 s after mixing. According to ASTM C1437 [48], the flow was tested, while setting time was measured via a modified Vicat needle according to ASTM C807 [49]. Accordingly, the effect of free water in the CAE on the hardening process of the H-AAM was investigated through the setting time tests.

#### 3.2.2. Mechanical Strength Tests

The compressive and flexural strengths were investigated based on BSEN 196-1 [50]. The mortar specimens were shaped into prisms measuring 40 mm × 40 mm × 160 mm. They were cured at ambient temperature and wrapped to prevent moisture evaporation, then tested on days 7 and 28 with the 250 kN–universal compression testing machine (UTM) of Controls-65-L12L2 Model (CONTROLS S.p.A., Milan, Italy). The force rates for the compressive and flexural strength tests were 2.4 kN/s and 0.5 kN/s, respectively. The two specimens were examined for flexural strength. The four halves of these failed prisms were tested continuously for compressive strength. The average strengths were recorded for each mixture.

#### 3.2.3. Water Absorption and Air Voids Tests

The water absorption and air voids were evaluated based on ASTM C642 [51]. Before the test, the prism-shaped specimens should be air-cured for 28 days. Each test sample was kept in the oven at 105 °C for 24 h and cooled in dry air over the next 24 h. Then the oven-dried masses were measured. They were next immersed in water for not less than 48 h, then the surface-dry mass in the air was recorded after they were removed from the water. At this point, the water absorption was calculated according to Equation (1).

(1) $$\mathrm{Absorption}(\%)=\frac{\left(W_{\mathrm{im}}-W_{\mathrm{oven}}\right)}{W_{\mathrm{im}}}\times 100$$

where Absorption (%) = water absorption of the specimen (%), W_oven_ = mass of oven-dried sample in the air (g), and W_im_ = mass of surface-dry sample in the air after immersion (g).

#### 3.2.4. Toughness Characteristics Definitions

The toughness, i.e., the ratio of the flexural to compressive strength, was used to describe the energy absorption of the material. It was calculated by using the data from the mechanical strength tests conducted on days 7 and 28.

#### 3.2.5. X-ray Diffraction Analysis (XRD)

The mineral compositions of the tested pastes were analyzed using XRD (Rigaku SmartLab, 10–50 2θ, Applied Rigaku Technologies, Inc., Austin, TX, USA). A single crystal X-ray diffractometer was used to capture the XRD patterns of 28-day-old pastes in this study.

#### 3.2.6. Scanning Electron Microscope Analysis (SEM)

SEM (JEOL JSM-5910LV, gold coated, WD: 11 mm, EHT: 15 kV, JEOL Inc., Pleasanton, CA, USA) was used to observe the pastes’ microstructures. Harden specimens were coated with gold before the examination.

#### 3.2.7. Fourier Transform Infrared Spectroscopy Analysis (FTIR)

The functional groups of the chemical compound present in the samples were examined with FTIR (Thermo Nicolet 6700, 4000–400 cm^−1^, Thermo Fisher Scientific Inc., Waltham, MA, USA). The 28-day-old pastes were ground into powder smaller than 75 microns and mixed with KBr powder. FTIR spectrometer was used to record their spectra information as percentages of transmittance and wavenumbers.

#### 3.2.8. Pore Distribution Analysis

The surface area analysis was conducted using the Brunauer, Emmett, and Teller (BET, Quantachrome Autosorb 1 MP, N_2_ gas, 99 points Adsorption–desorption, Anton Paar GmbH, Graz, Austria) method [52] on the adsorption isotherm. The pore size distribution was determined from the desorption isotherm via the Barrett, Joyner, and Halenda (BJH) method [53]. The Nitrogen adsorption and desorption isotherms were measured at 77.35 K while using a Quatachrome Nova station to test the 28-day-old pastes with volumes of 1 cm^3^. Prior to this test, each sample was degassed at 105 °C for 5 h.

## 4. Results and Discussion

### 4.1. Flow and Setting Time Tests

**Figure 5:**
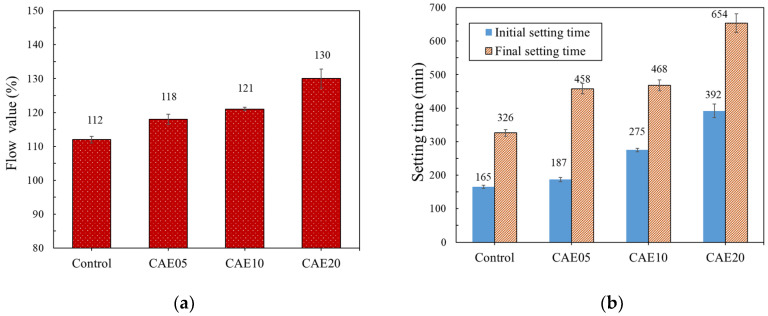
Results for the hybrid alkali-activated mortars with different CAE contents in terms of (**a**) setting times and (**b**) flow values.

Figure 5 shows the test flow values and setting times of mortars with different CAE contents. The results indicate that the hybrid alkali-activated mortar’s initial and final setting time values were significantly delayed with an increase in CAE content—as was true for the flow values. The mortar with 20% CAE provided the highest flow value, which was much larger than the value for the control mortar, and the setting time of this mortar was approximately twice that of the control mortar. The increase in flow was due to the extra water, which was 35% by the total amount of the CAE. From Figure 5a, the mortar with CAE contents of 5 and 10% had 187 and 275 min initial setting times, respectively. The final setting values were 458 and 468 min, respectively, both higher than 326 min recorded for the control mortar.

From Figure 5b, these results indicated that adding CAE could increase the flow values of the mixture. The CAE enhances the fluidity of the paste by providing more water for the mixing matrix through its demulsification. CAE can provide this extra water due to its free water content of approximately 35%. The emulsifier and excess water from the CAE accumulate in the free water according to the water-to-binder ratio, resulting in the excess water for the hydration of the pozzolanic materials, thus a prolonged hardening process during mixing. The H-AAM and CAE composite behaved like that of the cement-AE material and revealed the retardation effect of CAE, mainly caused by the high liquidity of the mixtures [24,25]. Hence, the addition of CAE can enhance the properties of this H-AAM remarkably.

### 4.2. Mechanical Properties

**Figure 6:**
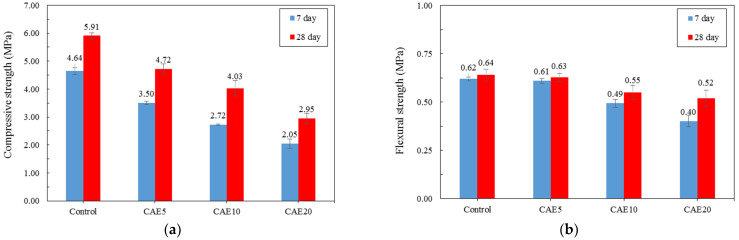
Results for the hybrid alkali-activated mortars with different CAE contents in terms of (**a**) compressive strength and (**b**) flexural strength.

Figure 6a illustrates the compressive strength of the test samples with different CAE contents. A higher CAE content resulted in a lower compressive strength. With more free water available with a higher CAE content, the water-to-cement ratio of the mixture increased, leading to a decrease in the overall compressive strength of the material. Furthermore, asphalt droplets in the mixture can cover pozzolan particles, delaying the reaction and forming weak spots [54,55]. In terms of flexural strength, Figure 6b shows the relatively small decline in flexural strength with higher CAE content for both seven- and 28-day curing periods. Note the relatively small gaps in flexural strength between the seven- and 28-day curing periods, regardless of CAE content. It seems that the CAE content did not play a leading role in developing flexural strength in the materials.

### 4.3. Water Absorption

**Figure 7:**
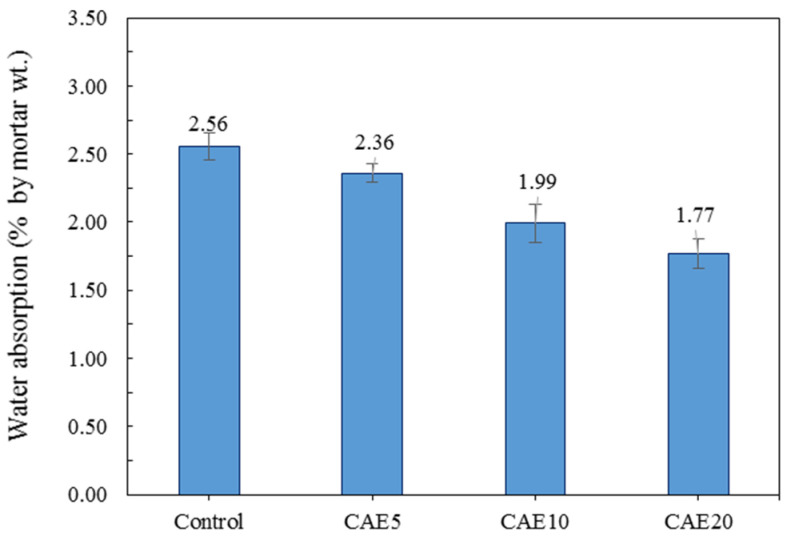
Water absorption values of the hybrid alkali-activated mortars with different CAE contents.

Figure 7 displays the results for the water absorption of the H-AAM mortars with different CAE contents. More CAE in the hybrid- alkali-activated matrix could reduce water absorption since the asphalt droplets in the CAE can cover and partly fill in the paste matrix, resulting in a barrier to water immigration along the mortar’s entire surface [56]. It can be explained by the presence of CAE-induced asphalt droplets in the matrix plugging in the free space between the binder and sand, resulting in impermeable space [37]. Furthermore, fewer air voids in the material matrix can lead to a considerable reduction in air porosity, resulting in improved surface barriers, the coated surface by the asphalt binders to protect the cementitious subtracts’ water consumption, to contact with water caused by the hydrophobicity of the emulsified asphalt coating [34,57].

### 4.4. Toughness Characteristics

**Figure 8:**
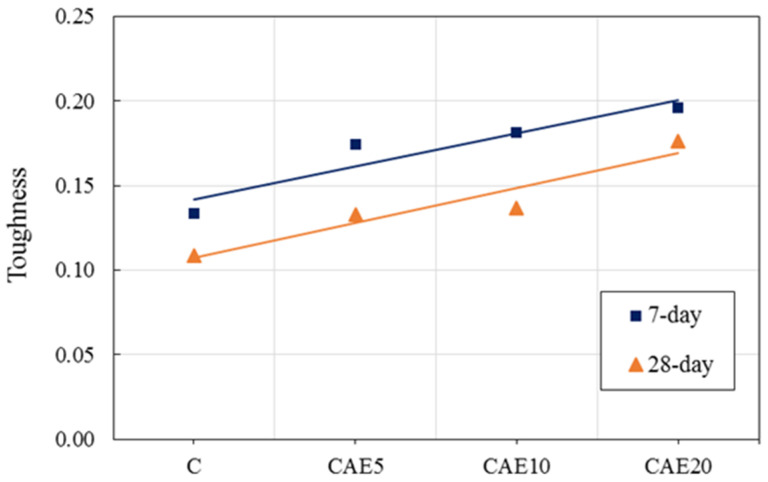
The linear lines of flexural-to-compressive strength ratio.

Figure 8 reveals the toughness characteristics of the H-AAMs with varying CAE contents. Note that adding CAE can increase the toughness of the hybrid alkali-activated mortars, with the highest CAE contents resulting in the most outstanding toughness. A material’s toughness generally indicates the brittleness and ductility of the material. Relatively higher material toughness implies flexibility, and the lower one indicates brittleness. Based on the toughness results exhibited in Figure 8, the presence of CAE in the H-AAMs provides ductility. These results agree with previous research, which indicated that more flexibility is present in polymer-modified cement-based materials [58,59]. Notably, more excellent material toughness indicates greater energy absorption, which is required for road pavement materials [60,61]. Therefore, CAE can generate better energy absorption behavior in the H-AAM, making it an ideal flexible road pavement material.

### 4.5. SEM Analysis

**Figure 9:**
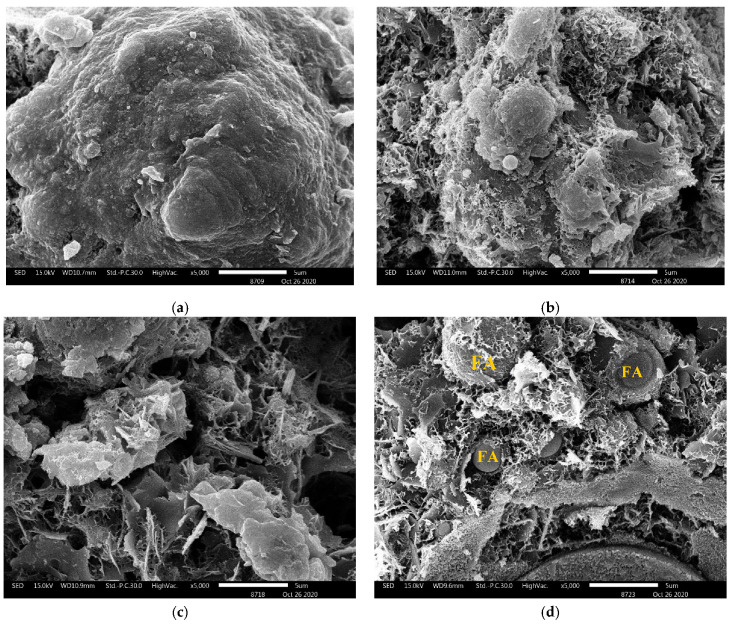
The SEM images of the pastes with different CAE contents in terms of (**a**) control, (**b**) CAE05, (**c**) CAE10, and (**d**) CAE20.

Figure 9 presents the morphologies of the hybrid alkali-activated pastes with different CAE contents obtained through the SEM analyses. Figure 9a shows the relatively smooth and continuous texture of the hybrid alkali-activated paste as a benchmark, indicating the large pieces of the solid cementitious product of the C-S-H gels. Adding CAE to the hybrid alkali-activated matrix did affect the texture. In Figure 9b–d, it is seen that a CAE layer surrounds the cluster of relatively smaller FA and HL particles. At a magnification of 5000×, agglomeration of material was found in control with 20 microns. Small sizes of particles were seen 5–10 microns with the increase in CAE content. It has been reported that different reaction products, including C-S-H gel, AFt, and CH were formed in paste without CAE, and pores were filled by the hydration products [35]. With CAE, asphalt film generated on the surface of fly ash particles, retardation of reactions occurred, and small sizes of fly ash particles were observed.

This clustering pattern of the CAE-coated particles indicates that the CAE addition did interrupt and retard the continuous cementitious product formation due to the pozzolanic reactions and alkali activations. Some unreacted FA particles are surrounded by CAE film layers connected to the hybrid alkali-activated paste. The microstructures indicate that more CAE contents had retarding effects on the chemical reaction needed for the hybrid alkali-activated formation. Furthermore, the 20% CAE addition provided a smoother consolidation in the matrices than lesser amounts of CAE due to CAE’s emulsifying property. In addition, CAE film, which was interlocked with and surrounded the hybrid alkali-activated particles, can plug and reduce some void spaces in the material matrix, resulting in the mortar’s lowest water absorption rate.

CAE layers covered the hybrid alkali-activated binder. These CAE layers governed the overall flexural resistance of the material. However, these CAE layers also played a vital role in the compressive strength reduction observed as the CAE content increased (see Figure 6). The thicker CAE layers produced by more CAE contents provide lower compressive load resistance.

### 4.6. XRD Analysis

**Figure 10:**
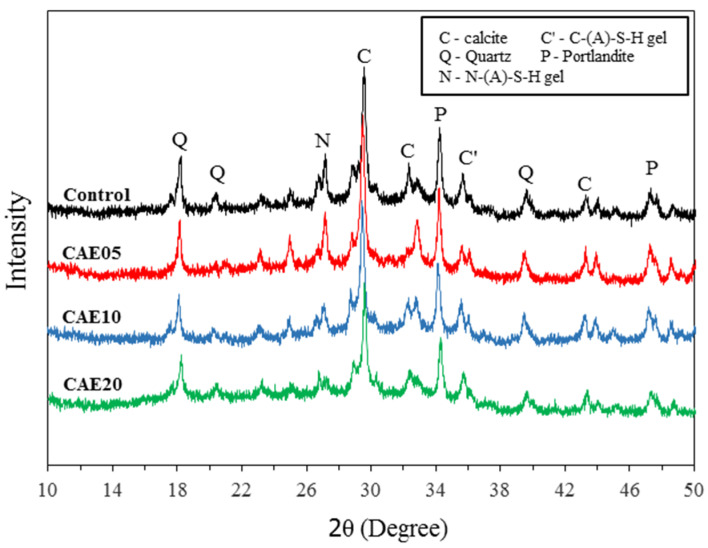
The XRD patterns of the hybrid alkali-activated pastes with different CAE contents.

Figure 10 illustrates the XRD patterns of the H-AAMs after 28 days of curing. The broad hump in the region of 26–38° 2θ was found in all samples and shows the amorphous phase of the material. The phases consist mainly of calcite, Portlandite, C-S-H, and N-(A)-S-H [9,12,14,62,63]. The control paste showed the peaks of C-S-H and N-(A)-S-H gels more clearly than the others with the CAE mixed in, indicating that the CAE retarded the alkali-activated reactions of the mixtures. Consequently, reductions in mechanical strength ensued. This result is similar to that obtained for the cement-AE system in previous research [42].

### 4.7. FTIR Analysis

**Figure 11:**
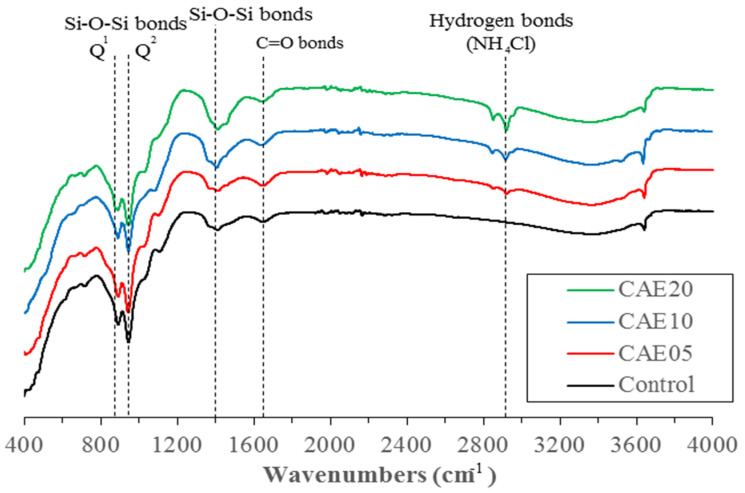
The FTIR spectra of the hybrid alkali-activated pastes with different CAE contents.

The FTIR results for the hybrid alkali-activated pastes with CAE contents are presented in Figure 11. Note that the intensities of the bands at about 835 and 960 cm^−1^ decreased as the CAE content increased. These two bands correspond to the asymmetric and symmetric stretching vibrations of the Si-O bonds, which can be considered part of the tetrahedrons in Q_1_ and Q_2_ from the structural formation of C-S-H [64,65]. The addition of CAE diminished gel formation. In contrast, the intensities of the bands at about 1375 and 2870 cm^−1^ increased as the CAE increased from 5 to 20% in the pastes. These wavenumbers refer to the stretching vibrations of the N-H bonds in ammonium chloride (NH_4_Cl), i.e., the emulsifier used in the CAE [66].

Furthermore, the C=O bonds indicated by the 1630 cm^−1^ band are from the hydrocarbon chains in the asphalt binder. Note that the band’s intensity gradually increases at the CAE addition increases from 5 to 20%. Based on these FTIR results, it is confirmed that the CAE did not react chemically with the pozzolanic material and AAMs. Hence, the FTIR results can validate CAE’s effect on this H-AAM in terms of obstructing the structural formations harmful to the material’s strength characteristics, as in the cement-based materials [38,67].

### 4.8. Pore Structure Analysis

Table 4 shows the results of BET surface area and total pore volumes following BJH pore size distribution desorption. The surface area of the hybrid alkali-activated mixtures decreased with the addition of CAE. The H-AAM with the 20% CAE addition provided the lowest surface area and had a high average pore diameter due to the asphalt droplets in CAE occupying pore spaces in the hybrid alkali-activated matrix. CAE’s asphalt droplets and emulsifying molecules refine the pores and are inserted into the hybrid alkali-activated matrix’s free space. The newly tiny pores are perhaps aggregated, leading to an increase in the average pore diameter. Consequently, CAE prevents extraneous water from penetrating this H-AAM by reducing the total number of pores.

Overall, these BJH pore analyses support the mortar test and validate that the addition of CAE plays a vital role in improving the water resistance of the H-AAM. It refined the pores and reduced the total pore volume of the material. The mixture with the highest CAE content would be excellent in terms of water and moisture resistance since the CAE reduced the average size of the capillary pores.

## 5. Conclusions

This study investigated the influence of CAE on the physical properties and structural formation of a H-AAM. According to the results, the following conclusions can be made:

Augmentation with CAE content gradually prolonged both the initial and final setting times of the hybrid alkali-activated mortar as it increased from 0 to 20% of the weight of the binder. Moreover, the study material’s compressive and flexural strength decreased markedly as the CAE content increased, resulting in more free water and reaction barriers to the hardening process, as confirmed by the microstructural analyses conducted in this study.

The flexural to compressive strength ratio increased with CAE content up to 20%, and the toughness of the hybrid alkali-activated mortar improved. Enhancements in material flexibility are possible through the addition of CAE.

CAE hinders cementitious formation resulting from the pozzolanic reactions and alkali activations in the hybrid alkali-activated pastes. This study revealed decreases in the cementitious product’s peak in the XRD tests and the number of tetrahedrons detected in the FTIR tests. The SEM images showed some signs of asphalt films surrounding hybrid alkali-activated particles and even some unreacted FA particles, indicating incomplete chemical reactions in the study material’s matrix.

The water absorption of the hybrid alkali-activated mortar decreased with the CAE content from 0 to 20%. The CAE increases the average pore size of the hybrid alkali-activated paste while decreasing the surface area, hindering the cementitious formation of the study material.

Based on this study’s results, the H-AAM with a CAE addition could represent a sustainable construction material with water repulsion and flexibility, ideal for pavement base stabilization. This material should be subjected to further investigations concerning its environmental and economic benefits. The performance-based parameters such as resilient modulus, shrinkage, and cyclic tensile modulus are recommended for further studies of this new material to understand specific pavement properties based on more realistic conditions.

## Figures and Tables

**Table 1:** Chemical compositions of the raw materials.

Chemical Composition (% by Total Weight)	FA	HL
SiO_2_	32.47	0.6
Al_2_O_3_	15.84	0.5
Fe_2_O_3_	14.64	0.1
CaO	24.83	74.5
SO_3_	4.3	-
P_2_O_5_	0.31	-
K_2_O	1.87	-
MnO_2_	0.2	-
TiO_2_	0.46	-
MgO	3	1.1
Na_2_O	1.99	-
LOI *	0.09	23.0

* LOI—loss of ignition.

**Table 2:** Properties of the CAE.

Property	Unit	Result
Density	(g/cm^3^)	
Storage (24 h)	%	1.2
Sieve (1.18 mm)	%	0.00
Particle charge		Positive
Solid content	%	67.3
Softening point, °C	°C	61.7
Elastic recovery at 25 °C	%	64
Penetration at 25 °C	mm	65
Ductility at 25 °C	cm	95
Solubility	%	99.34

**Table 3:** Mortar mix design used in this study.

Mix	Material Proportions (% of Binder’s Weight)				w/b	b/s	a/b Ratio
	FA	HL	SH	CSS-1h			
Control	77.3	19.3	3.4	-	0.45:1.00	1.00:2.75	-
CAE 05	77.3	19.3	3.4	5.0			0.05
CAE 10	77.3	19.3	3.4	10.0			0.10
CAE 20	77.3	19.3	3.4	20.0			0.20

**Table 4:** BET surface and total pore volume results.

Mixture	Average Diameter	Surface Area	Total Pore Volume
	(nm)	(m^2^/g)	(cm^3^/g)
Control	35.752	12.705	0.114
CAE05	39.481	10.107	0.112
CAE10	44.603	9.520	0.094
CAE20	48.812	6.955	0.085

## Data Availability

The data presented in this study are available on request from the corresponding author. The data are not publicly available due to a restriction in the funding agreement.
